# Social vulnerability assessment under different extreme precipitation scenarios: A case study in Henan Province, China

**DOI:** 10.1371/journal.pone.0299956

**Published:** 2024-03-08

**Authors:** Xiaobing Zhou, Yongling Zhang, Wei Wang, Xin Li, Huanhuan Yang, Yiting Sun

**Affiliations:** 1 School of Business Administration, Henan Polytechnic University, Jiaozuo, China; 2 School of Emergency Management, Henan Polytechnic University, Jiaozuo, China; 3 Security and Emergency Management Research Center, Henan Polytechnic University, Jiaozuo, China; Qufu Normal University, CHINA

## Abstract

Extreme precipitation usually cause grievous losses&casualties, which varies greatly under different scenarios. This paper took Henan province as an example, it innovatively constructed three different extreme precipitation scenarios and built indicators system of social vulnerability from exposure, sensitivity and resilience based on MOVE framework. Social Vulnerability Indexs(SoVI) were then calculated by mathematical models under three different reoccurrence intervals. The results show that SoVI was low in the west and high in the north. High SoVI areas expanded to the middle and south as recurrence intervals increased. SoVI in each area of Henan province increased along with the recurrence intervals at different growth rates. The larger the recurrence interval was, the faster the SoVI increased. The results indicate SoVI is greatly affected by disaster levels, which need to be incorporated into social vulnerability. This study provides not only a new thought for social vulnerability assessment, but also a reference for the policymakers to formulate related risk management policies.

## 1. Introduction

Extreme precipitation is one of the most common and devastating events causing nearly half of all victims of natural disasters [1–3]. According to the Six Assessment Report of IPCC (AR6) ‘Climate Change 2021:The Physical Science Basis’, In the future several decades, climate change all over the world will aggravate, one of the results is that extreme precipitation event will be more and more frequent. Reported by McKinsey report, the kind of heavy precipitation that was a once in 50-year event in 1980 is expected to be two to three times more likely in 2030 and three to six times more likely in 2050 in China. The population affected is likely to continuously increase and economic losses may double. Besides, climate change is influencing the rainfall distribution. Precipitation is likely to increase in high latitude areas and decrease in most subtropical areas. As for China, the temperature increase is faster than global average. Extreme precipitation disasters increase both in frequency and intensity, which pose a more and more threat to human life and property. Based on the trend of climate change and discrepancy of vulnerabilities and exposures in different areas, IPCC (AR6) estimates that East China which is densely populated and economic aggregated is at high risk of extreme precipitation and will bear high climate risk.

Displayed by Global Disaster Data Platform, China ranked first in extreme precipitation disasters with frequency of 96, affected population 240 million and direct economic loss RMB115.446 billion during last decade. Especially in June 2020, altogether 29 provinces, 290 municipalities and 1904 counties were hit by extreme precipitation. A large population of 7.37×10^7^ were affected, 278 persons died or disappeared and caused direct economic loss RMB 220 billion, which respectively occupied 47% of the deaths or missing persons and 59% of economic losses caused by natural disasters of that year [4]. Recently, extreme climate variability, extended urbanization, poor facilities and management make it one of the most flood-prone provinces in China [5, 6]. Recently, affected by climate change, extreme precipitation attacks Henan province more and more frequently and seriously. Especially in July, 2021, Henan province was subjected to an extraordinary rainstorm, with the average precipitation exceeding the recorded extremum greatly. According to the investigation report issued by Disaster Investigation Team of State Council, around 1.48 ×10^7^persons in 150 counties were affected by the rainstorm, causing 398 casualties, among which 380 in Zhengzhou, 10 in Xinxiang, 2 in Pingdingshan, Zhumadian and Luoyang respectively, 1 in Hebi and Luohe separately. The direct economic loss was RMB120.06 billion, among which, Zhengzhou, the capital of Henan province witnessed the worst with RMB 40.90 billion. Extreme precipitation attacked Henan province not only once. In August 1975, Affected by super typhoon, extreme precipitation swept Henan province, which triggered extraordinary floods and caused nearly 60 reservoirs collapsed. Totally 1.10×10^7^ persons in 29 counties were stricken, nearly1.13×10^10^ km^2^ farmlands were drowned, 5.96×10^6^ houses collapsed, 3.023×10^5^ farm animals were washed away. 102 kilometers of Beijing-Guangzhou Railwayline, which passes through China from north to south were destroyed. The total direct economic losses reached nearly RMB 10 billion.

Extreme precipitation disasters attack Henan province frequently, which lead to serious casualties and damages. How to prevent and mitigate the loss of extreme precipitation is both crucial and urgent. One of its management strategies is to reduce the disaster risk [7]. Vulnerability assessment is an essential component of disaster risk management [8]. This paper took Henan province as an example and made an assessment on the social vulnerability to extreme precipitation under reoccurrence intervals of 50a, 100a and 200a to reveal its spatial distribution and evolution laws of social vulnerability, hoping to provide a new and practical approach for disaster mitigation and risk reduction of extreme precipitation.

## 2. Literature review

In theoretical perspective, Birkmann [9] pointed out that different views on vulnerability could be classified in different schools of thought that include: school that analyzes the vulnerability from both internal and external perspectives: internally as the ability to anticipate, address and recover from the impact of a disaster in contrast to the external perspective, which involves the degree of exposure to such threats. It illustrates how vulnerability results from a combination of internal and external processes, including the system’s underlying susceptibility and defenselessness, as well as external risks, shocks, and stresses. School of Disaster Risk Reduction (DRR) sees vulnerability and disaster risk assessment from a holistic view and defines vulnerability as a component of disaster risk, differentiating exposure and responsiveness. Song [10]suggested incorporating the concept of adaptation as an element that increased resilience and integrating adaptation and coupling processes into a feedback-loop system based on process-oriented perspective of vulnerability. Eriksen et al. [11] developed the concept from the political economy, seeking the causes, dynamic pressures and unsafe conditions that determined vulnerability. Recently, with acceleration of climate change and frequent extreme weather phenomenon, school of vulnerability has emerged that combines framework of disaster risk research and climate change adaptation which focuses on exposure, sensitivity and adaptive capacity in urban areas as key determinants of vulnerability.

Though many definitions exist, this study adopts the concept provided by Abdur Rahim Hamidi [12] who deemed that vulnerability to natural disasters were different for a population or a system with different conditions depending upon three significant aspects, i.e., exposure, susceptibility and resilience. Exposure referred to the nature of the stimuli (e.g. extreme precipitation) and its characteristics (e.g., magnitude, spatial extent, frequency, duration). Sensitivity can be defined as the predisposition of elements at risk to suffer harm [13]or ‘a tendency/degree of elements at risk’ that can come to any harm as a result of the disaster [9]. Resilience denoted the magnitude of disturbance that can be absorbed before a system changes to a radically different state as well as the capacity of the areas to self-organize and adapt to emerging circumstances [14]. It may be very difficult to control the exposure and sensitivity, but increasing adaptive capability coupled with structural and non-structural measures can minimize the impact of a natural disaster.

Vulnerability can be viewed as being both physical and social, and these two factors can interact in complex ways. Physical vulnerability relates to the external risk factors, the natural causes of disasters such as heavy rainfall [15]. While social vulnerability relates to social, economic, political and institutional conditions which influence susceptibility to harm and govern ability to respond to disasters [16]. Social vulnerability varies as a function of the research scale, the specificities of the disaster and the particular conception of vulnerability adopted, thus, conceptions of social vulnerability are largely scale dependent [17, 18]. From the existing literatures, social vulnerability can be understood from three scales: at macro-level, the researches mainly focus on global or national scale, related determinants affecting vulnerabilities are primarily socioeconomic status, educational level, occupation, demographic variables, persons with disabilities, housing quality, access to basic services, ethnicity/immigrants. Specific indicators such as relative mortality rate and relative GDP losses have also been used [19]. At medium-level, factors which may influence vulnerability in a region can be categorized in social, geotechnical, structural, and physical parameters. The degree of impact suffered from a harmful event is related to the regional risk and its social & economic conditions [20]. At micro-level, the local or community vulnerabilities are the starting point and objective, methodologies for assessing social vulnerability vary greatly under different context of analysis and the availability of data. Nevertheless, vulnerabilities are evaluated from three dimensions: exposure, susceptibility and adaptive capability. Relevant indices are typically composed of age, disabilities, income, occupation, race, family status, housing and infrastructure and lifelines [21, 22].

In conclusion, at macro-level or medium-level, the present literatures assess social vulnerability mostly from susceptibility factors [23–25], whereas exposure elements, the component of vulnerability determining the magnitude and level of risks is missing. Although exposure factors are usually considered at micro-level, they refer to the degree that people, assets and environment are exposed to disasters, which is different from exposure at macro-level or medium level in indicator selection. In view of this, this paper make possible innovations as follows:(1) It designed specific extreme precipitation scenarios and calculated the extreme precipitations accordingly, which is seldom seen in current social vulnerability assessment at macro-level that only depends on statistic data of the Year Book, neglecting the specific scenarios that extreme precipitations occurs.(2) It introduced exposure that represents the precipitation level into the indicators system of social vulnerability assessment, considering the natural property of disasters.(3) It constructed three extreme precipitation scenarios under three recurrence intervals (50a, 100a, 200a) and assessed the social vulnerability accordingly, thus to reveal the evolvement rules of social vulnerability to extreme precipitation.

Three questions form the basis of this work: faced with different recurrence intervals of extreme precipitation, will the social vulnerability of each areas change or not? If yes, how is the change? And what are the spatial distributions and evolution laws of social vulnerabilities in Henan province under different recurrence intervals? To address these questions, this paper firstly constructed a set of indicators from exposure, sensitivity and resilience based on MOVE framework and set three different scenarios of extreme precipitation, based on which, social vulnerability index (SoVI) to extreme precipitation in Henan province were calculated and mapped. The approaches and results presented in this study will help local governments to identify which subsystems of the society are more susceptible to extreme precipitation risks and develop effective risk reduction strategies that are tailored to local conditions.

## 3. Studying area and evaluation methodology

### 3.1 Studying area

Henan province lies in the center of China, covering an area of approximately 1.67×10^5^ km^2^. The terrain is high in the west and low in the east, which consists of plains, basins, mountains, hills and water surfaces. Most of the province is located in the warm temperate zone, with the southern in subtropics. Influenced by monsoon, precipitation between different areas are uneven and vary greatly in different years even in the same area. Furthermore, precipitation is nonuniformly distributed among seasons with the most in summer.

Henan province has jurisdiction over 18 administrative areas with a large population of 9.9365519×10^7^ permanent residents by November 2020, ranking third in the nation according to the results of the Seventh National Census, among which, 55.43% dell in urban areas, leading to a increasing urbanization rate of 56.45% by the end of 2021.

Why we choose Henan province as study area is that Henan province is the main force of Central Plains Economic Region and important grain production base. Due to the extreme climate variation, quick urbanization, unreasonable industrial structure, poor infrastructure and resilience, it is frequently invaded by extreme precipitation, which highlight the need to evaluate its social vulnerability and identify the hotspots or vulnerable people and place, thus to take measures to avoid or mitigate losses caused by extreme precipitation.

### 3.2 Data and methodology

#### 3.2.1 Evaluation units

This study made a research on the social vulnerability to extreme precipitation in Henan province under different reoccurrence intervals, selecting 18 administrative areas as evaluation units.

#### 3.2.2 Development of social vulnerability index

**Fig 1:**
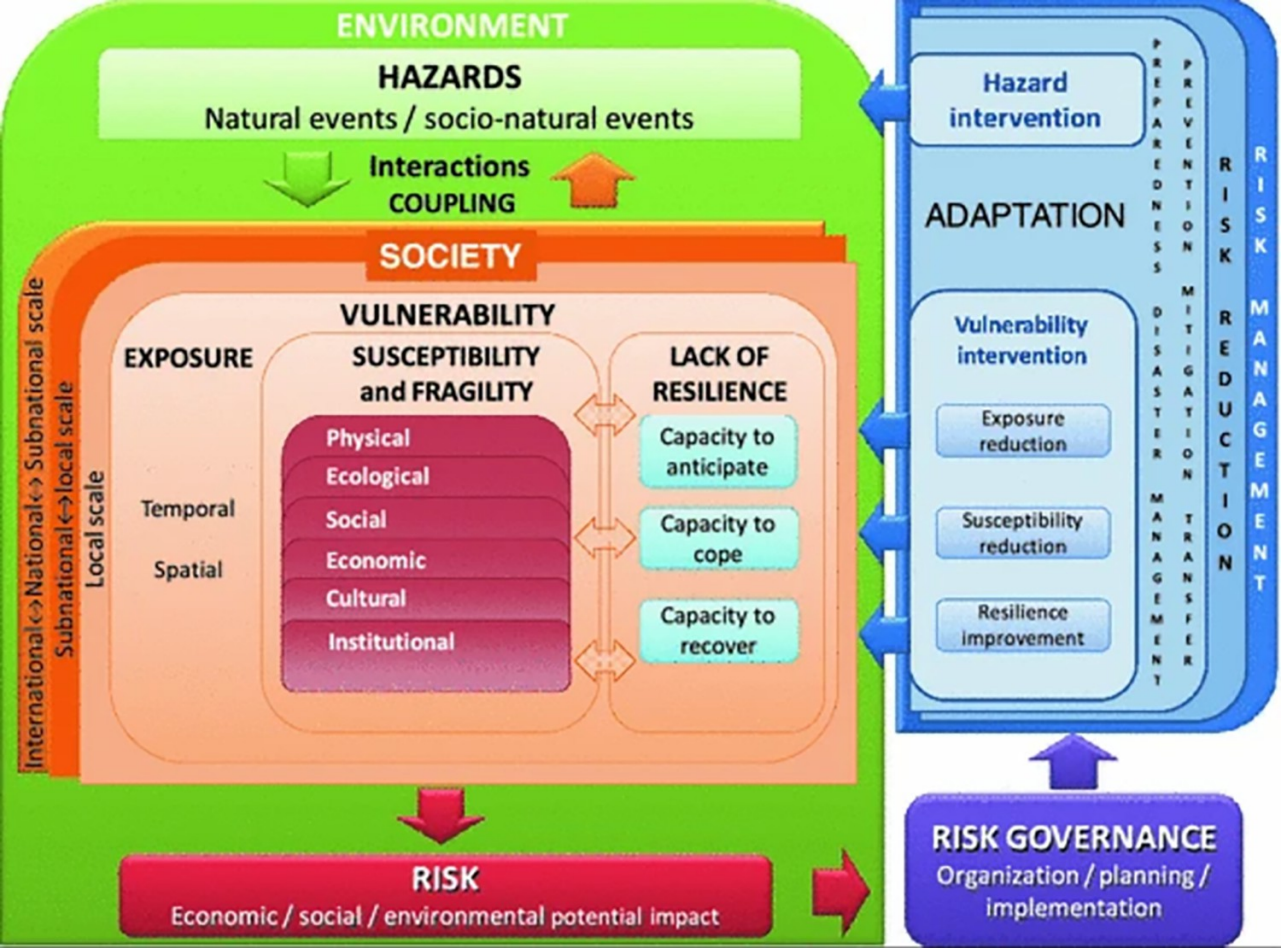
MOVE framework. (Extracted from Birkmann et al. 2013).

*(1) Framework of social vulnerability index*. A model is vital for measuring vulnerability, as it provides structure and framework for the development of rainstorm vulnerability indicators [26]. Plenty of approach/model are available in the literature to assess vulnerability according to different schools of thoughts, such as ‘political economy’, ‘social–ecology’, ‘holistic vulnerability’, ‘climate change science’, ‘HOP, ‘PEOPLE’, ‘BBC’, ‘MOVE’ [27]. Among them, MOVE framework is a ‘heuristic thinking tool’ that underlines significant factors and dimensions of vulnerability, it integrates exposure, susceptibility and resilience in vulnerability as key underlying factors and defines vulnerability as exposure of a system to disaster, its level of susceptibility and coping capability or resilience. Moreover, MOVE framework also emphasizes the significance of considering various thematic dimensions of natural and societal aspects such as physical, socioeconomic, environmental and institutional, making it more relevant to vulnerability assessment. Thus, the present study used MOVE framework to provide guidance and criteria for developing indicators (see Fig 1).

*(2) Selection of social vulnerability index*. Social vulnerability can be distributed into two parts: the vulnerability of the disaster-bearing body and the response capability of disaster relief [28]. The former refers to such as population size, population density, gender, age, income and family structure. Specific group of people are more vulnerable such as elderly persons and single-parent families. Females have responsibility to look after family members and take up all family matters, which make them more vulnerable [29]. The latter mainly refers to the resilience of the place to prepare for, mitigate, cope with and recover from disasters with structural and nonstructural measures. In addition, exposure to what degree of disaster will influence the coping or adaptive capability against disaster and consequently affect vulnerability. The influencing factors are numerous, by referring to literatures and consulting with experts, simultaneously considering the local context of Henan province and the availability of data, this paper constructed social vulnerability indicators system from exposure, sensitivity and resilience based on MOVE framework, which incorporated 18 indicators. In views of the strong correlation between some indicators, Pearson correlation analysis was made. If the correlation coefficient between two indicators was greater than 0.8 or less than -0.8, one of the indicators would be retained at random. Finally, 14 indicators were retained (see Table 1).

**Table 1:** Indicators system of social vulnerability to extreme precipitation in Henan province.

Indicator dimensions	Indicators	NO.#	Weight of indicators(%)	Functional correlation of indicators
EI	Day extreme precipitation under 50a/100a/200a	X1	20.457	+
SI	Population density	X2	12.004	+
	Female proportion	X3	1.390	+
	Proportion of old and young	X4	3.136	+
	Investment growth rate of industrial fixed assets	X5	4.386	+
RI	Average number of students in high school and above every ten thousand people	X6	1.644	_
	Number of heath institutions per unit area	X7	5.033	_
	Number of hospital beds every ten thousands people	X8	5.839	_
	Average disposable income of residents	X9	2.433	_
	Drainage network density of build up area	X10	16.982	_
	Number of reservoirs	X11	8.971	_
	Highway passenger volume every ten thousands people	X12	1.880	_
	Density of community service institutions	X13	8.850	_
	Proportion of expenditure on public safety	X14	6.996	_

Note: EI, SI, RI respectively means exposure index, sensitivity index and resistance index. The data source from Henan Statistical Year Book 2020.

#### 3.2.3 Data sources and processing

*(1) Data sources*. Data of sensitivity indicators and resilience indicators were collected from Henan Statistical Year Book 2020 issued by Henan province Bureau of Statistics (https://tjj.henan.gov.cn/tjfw/tjcbw/tjnj/). Data of exposure indicators were obtained by using Pearson-Ⅲ distribution method to calculate day extreme precipitation on Microsoft’s Excel 2000 under three different recurrence interval (50a,100a,200a). The original data were acquired from observation stations from 1991 to 2021 in National Weather Service Meteorological Data Center (http://data.cma.cn/).

*(2) Data processing*. As the social vulnerability indicators involved various unit of measurement, the range of the value differed and varied greatly. The general minimum–maximum method was used for rescaling data that brought the indicator data to zero and one. Both exposure and susceptibility had a positive influence on vulnerability, and resilience had a negative influence on vulnerability. For the indicators that had a positive relationship, the normalization was done using the formula given below:

$$xi^{\prime }=\frac{xi-minxi}{maxxi-minxi}$$ (1)

Where *xi*′ is normalized value. *xi* is the original value of the indicator *i*. After normalization, the scores of these indicators are made to lie between zero and one. In this condition, the greater value the indicator obtains, the higher vulnerability it owns, and vice versa. The summation of the two factors (exposure and sensitivity) that have positive relationship with vulnerability yield the higher vulnerability index.

On the contrary, indicators of resilience have a negative relationship with vulnerability, the normalized score was computed according to the formula as below:

$$xi^{\prime }=\frac{maxxi-xi}{maxxi-minxi}$$ (2)

#### 3.2.4 Weighing indicators

At present, the weight determination methods are mainly divided into subjective weighting method and objective weighting method. The former mainly includes expert scoring, hierarchical analysis, pairwise comparison method, etc. The latter primarily includes fuzzy mathematical method, entropy method, coefficient of variation method, et al. As one of the objective weighting method, entropy method fixes weight by judging the dispersion degree of the index based on the amount of information provided by the value of the indicators. The greater the dispersion degree is, the greater this index will influence on the comprehensive evaluation. To some extent, it avoids the defects of arbitrariness of subjective weighting method, so we adopted entropy method to weight the indicators based on the formulas as follows:

Compute the proportion of indicator j in year i

$$Yij=\frac{Xij}{\sum_{i=1}^{m}Xij}$$ (3)

Compute the comentropy of the indicator j

$$ej=-k\sum_{i=1}^{m}(Yij*\ln\;Yij)$$ (4)

Compute the redundancy of comentropy

dj=1−ej (5)

Compute the weight

$$wi=dj/\sum_{j=1}^{n}dj$$ (6)

Where *Xij* indicates the value of indicator j in year i, K = 1/ln *m*, m is the number of years, n is the number of indicators.

#### 3.2.5 Constructing model of social vulnerability index

According to the built indicators system (Table 1), social vulnerability to extreme precipitation in Henan province can be assessed from three dimensions: exposure index(EI), sensitivity index (SI) and resilience index(RI), we defined the computing formulas as follows:

$$EI=\sum_{i=1}^{1}xi^{\prime }\cdot wi$$ (7)

$$SI=\sum_{i=2}^{5}xi^{\prime }\cdot wi$$ (8)

$$RI=\sum_{i=6}^{14}xi^{\prime }\cdot wi$$ (9)

Where EI indicates exposure index, the higher the value is, the greater the social system is exposed to extreme precipitation. SI denotes sensitivity index, the higher the value is, the more sensitive the social system is. RI signifies resilience index, the higher the value is, the stronger ability the social system possesses to defense, cope with and adapt to extreme precipitation. *xi*′ denotes the standardized data of indicator i, *wi* means the weight of indicator i.

Based on the indicator system built above and the function correlation with social vulnerability, this study constructed the assessment model of social vulnerability index (SoVI), the computing formula was as follows:

$$SoVI=\frac{EI+SI}{RI}$$ (10)

Where EI, SI, RI, SoVI respectively indicated exposure index, sensitivity index, resilience index and social vulnerability index, the higher value the SoVI obtains, the greater vulnerability the society exists.

## 4. Results

### 4.1 The spatial distribution of EI, SI and RI in Henan province

#### 4.1.1 The spatial distribution of EI under three extreme precipitation scenarios in Henan province

**Fig 2:**
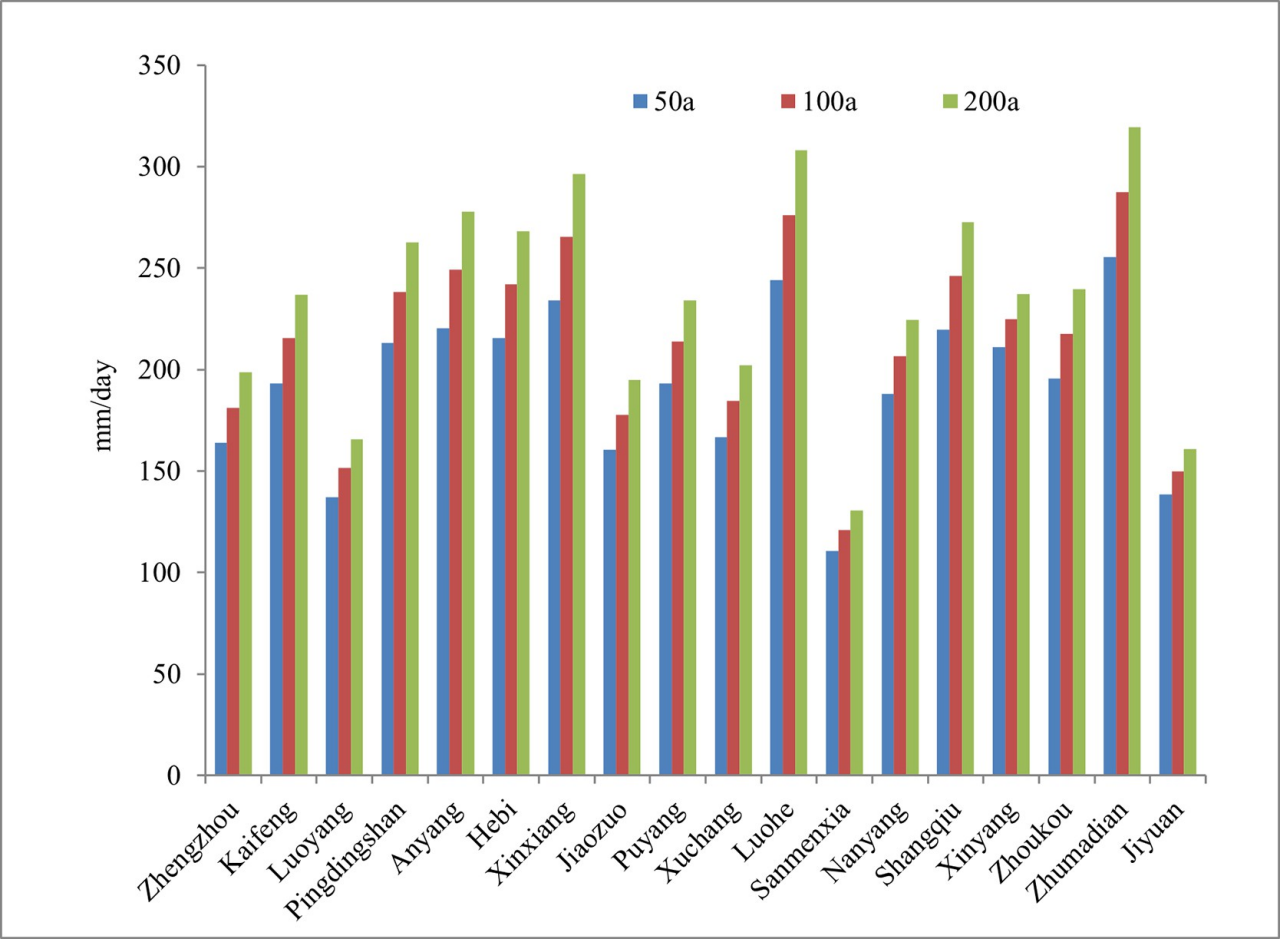
Variation trend of three extreme precipitation scenarios in each area of Henan province. (Drawn by the author themselves).

Fig 2. shows the variation trend of three extreme precipitation scenarios. As can be seen, extreme precipitation in each area increased obviously as the reoccurrence intervals enlarged, which indicated that the exposure risk magnified when reoccurrence intervals increased.

**Fig 3:**
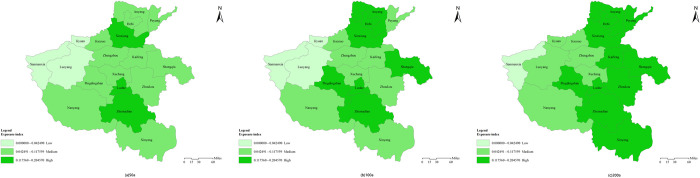
Spatial distribution of EI under three extreme precipitation scenarios in Henan province. (The author used ARCGIS10.2 to draw. The base map is based on the standard map GS(2017)1268 publicly provided by the Ministry of Natural Resources of P.R.C).

The spatial distribution of EI under three recurrence intervals in Henan province is shown in Fig 3. As it displays, most of Henan province were in high and medium exposure. EI was high in southeast and low in northwest. High exposure areas increased and low exposure areas decreased as the recurrence intervals enlarged. To be specific, under recurrence intervals of 50a, Xinxiang, Luohe, Zhumadian were in high exposure areas since they were respectively in the watershed of Shahe&Yinghe, Huanghe&Haihe and suffered large day max precipitation. Jiyuan, Luoyang, Sanmenxia which lied in western mountain areas were low in EI. High exposure areas increased by four areas under 100a, namely: Anyang, Hebi, Shangqiu and Pingdingshan, low exposure areas remained the same as 50a. When the reoccurrence intervals increased to 200a, high exposure areas increased synchronously by four areas(Puyang, Kaifeng, Zhoukou, Xinyang) compared with 100a, while the low exposure area decreased to one: Sanmenxia, which had the least extreme precipitation.

#### 4.1.2 The sensitivity index under extreme precipitation in Henan province

**Fig 4:**
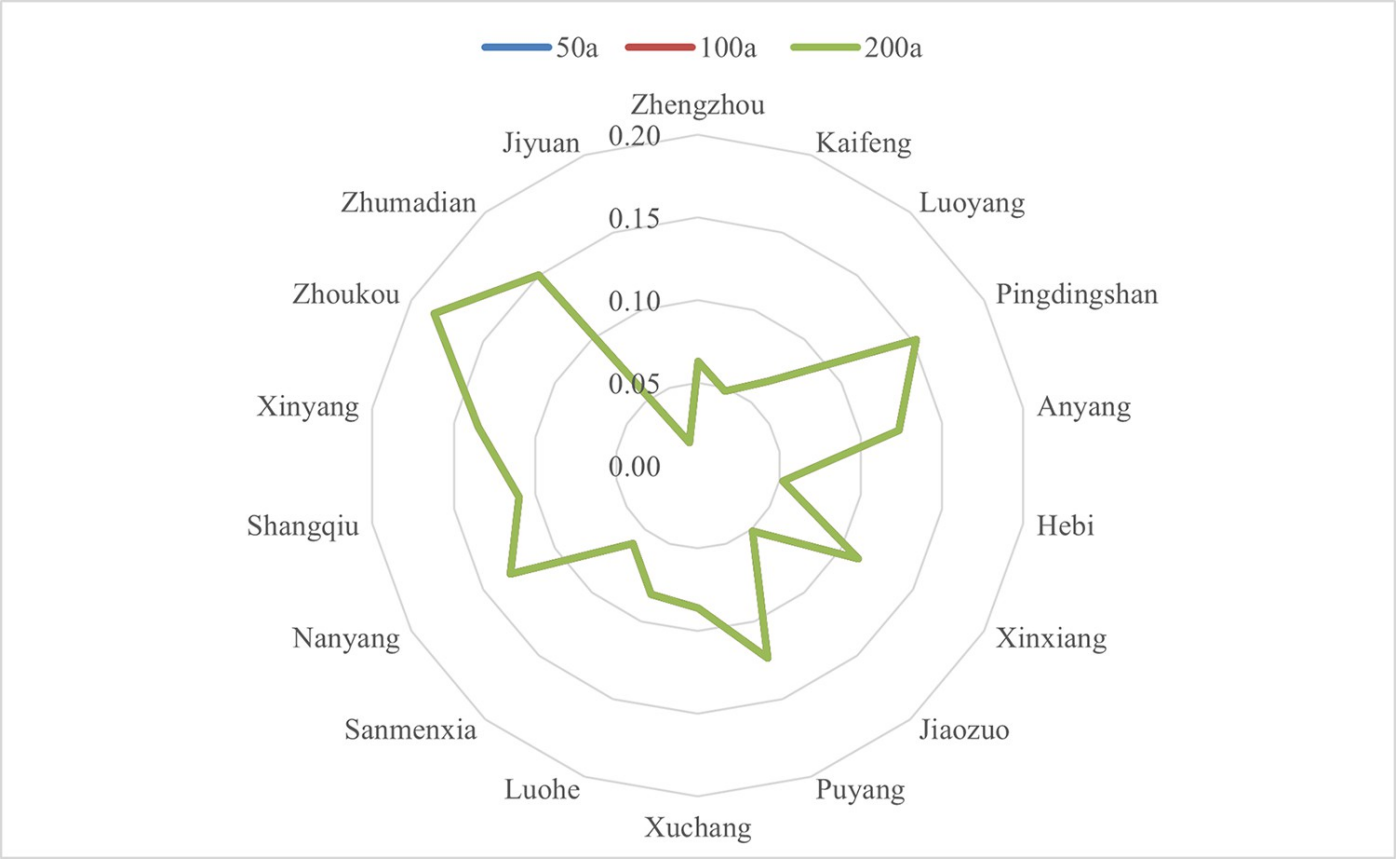
Radar image of sensitivity index under extreme precipitation in Henan province. (Drawn by the authors themselves, the numbers in the figure are the sensitivity index in each area).

The sensitivity index under extreme precipitation in Henan province is shown in Fig 4. As was seen, the sensitivity index under three recurrence intervals coincided, Jiyuan, Jiaozuo, Hebi and Kaifeng were low sensitive areas, as these areas had a relatively low population density as well as comparatively developed economy. Zhoukou, Pingdingshan and Zhumadian were high sensitive, which owned to not only the large density of population, but also higher old &young proportion(around 40%, far more than the average 36.4%) and higher investment growth rates of industrial fixed assets.

#### 4.1.3 The resilience index under extreme precipitation in Henan province

**Fig 5:**
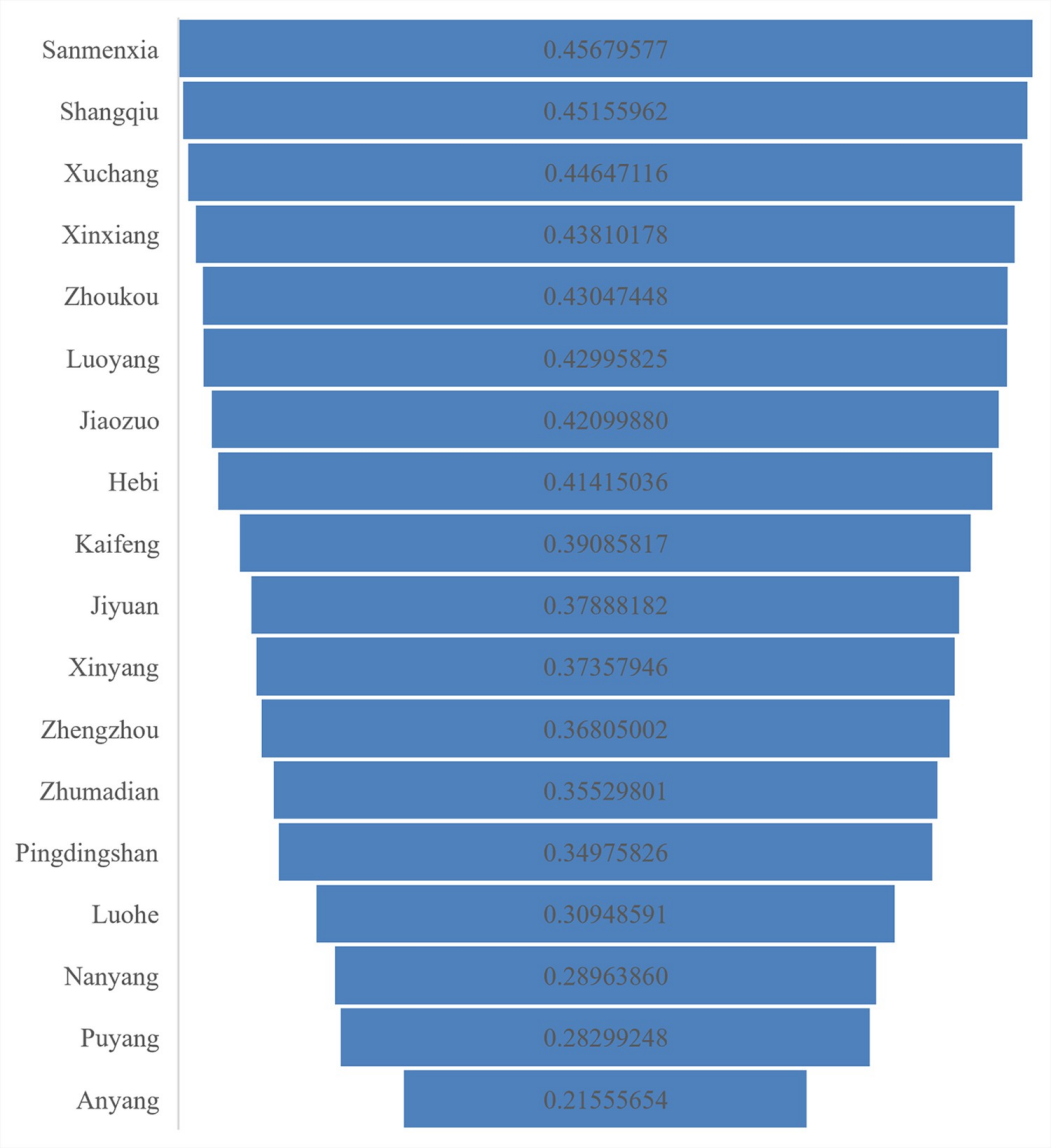
The resistance index under extreme precipitation in Henan province. (Drawn by the authors themselves, the numbers in the bar are the resistance index in each area).

Fig 5 displays the resilience index under extreme precipitation in Henan province. As is revealed, Anyang, Nanyang, Luohe and Puyang were low resilient regions due to low GDP per capital, drainage network density and few hospital institutions. In addition, the average number of students in high school in these areas were also lower than the average. High resilient areas were Shangqiu, Xuchang and Sanmenxia on account of the thorough infrastructures, especially, the highway passenger volume every ten thousands people and proportion of expenditure on public safety were both higher than the average.

### 4.2 The variation trend of SoVI and its spatial distributions under three recurrence intervals

**Fig 6:**
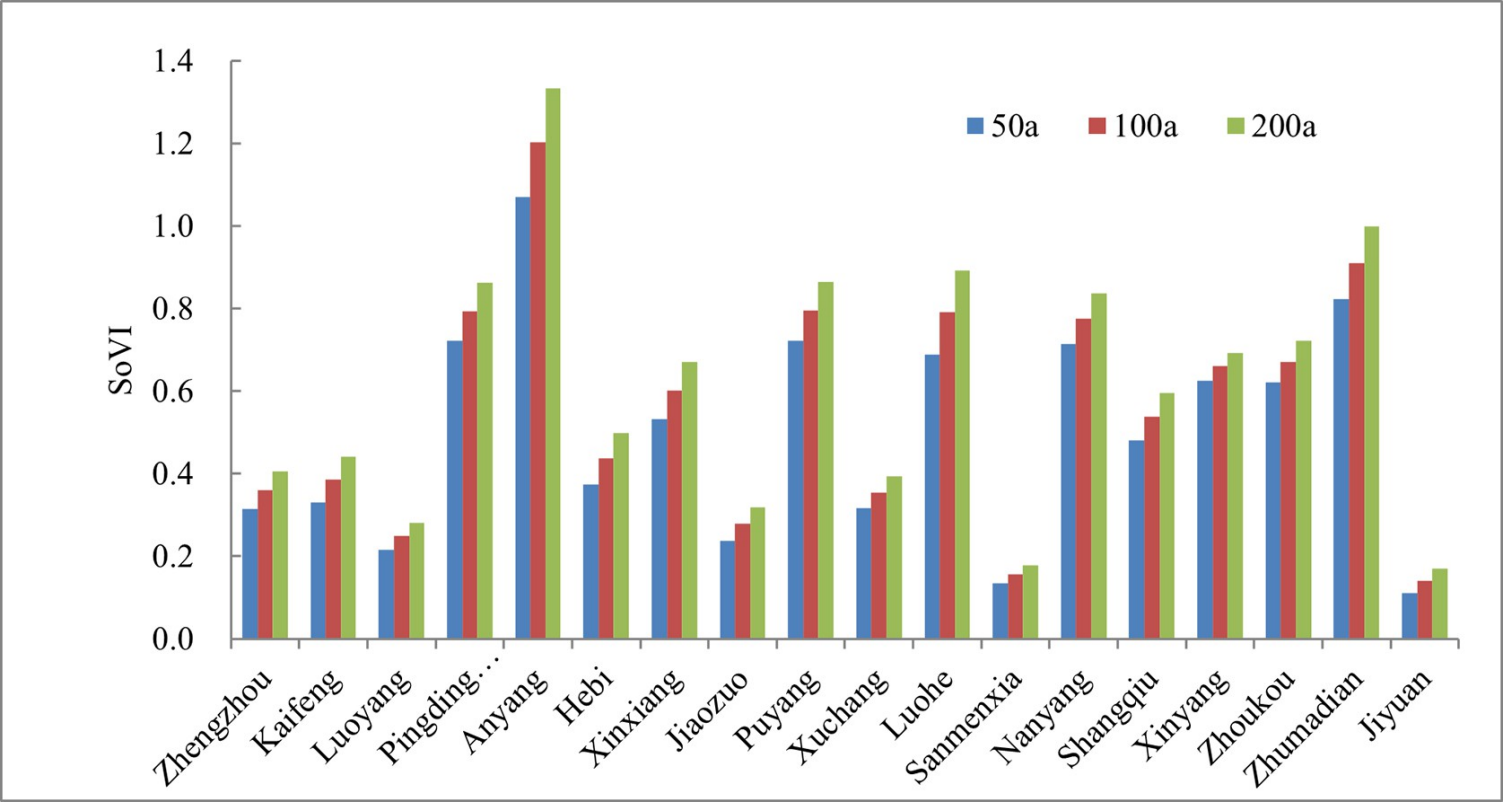
Variation trend of SoVI to extreme precipitation in each area under three recurrence intervals. (Drawn by the authors themselves).

The variation trend of SoVI in each area under three recurrence intervals is displayed in Fig 6. As was seen, SoVI in each area of Henan province was widely different. Jiyuan, Sanmenxia was the lowest in SoVI, followed by Luoyang and Jiaozuo. Anyang was the highest in SoVI, Zhumadian, Pingdingshan and Luohe took second place.

**Fig 7:**
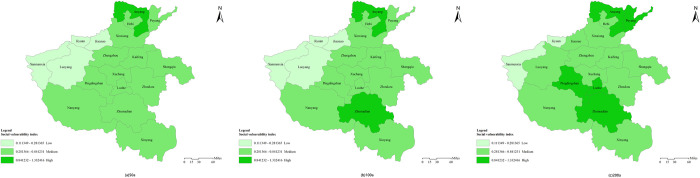
Spatial distribution of SoVI to extreme precipitation in Henan province under three recurrence intervals. (The author used ARCGIS 10.2 to draw. The base map is based on the standard map GS(2017)1268 publicly provided by the Ministry of Natural Resources of P.R.C).

The spatial distribution of SoVI under three recurrence intervals in Henan province is shown in Fig 7. As it displays, the northern area was high in SoVI, high SoVI areas increased and low SoVI areas decreased as the recurrence intervals enlarged. In terms of recurrence interval of 50a, the SoVI presented low in the western areas, high in the northern areas and moderate in the remaining regions. To be specific, Anyang was the only high social vulnerability area. Jiyuan, Luoyang, Sanmenxia and Jiaozuo were low in social vulnerability. The remaining areas were medium in SoVI. When recurrence intervals enlarged to 100a and 200a, areas with high SoVI increased and low SoVI areas decreased gradually. High social vulnerability areas increased respectively to two (Anyang, Zhumadian) and five (Anyang, Zhumadian, Pingdingshan, Luohe and Puyang), low social vulnerability areas decreased to two (Jiyuan and Sanmenxia).

## 5. Discussion

This study assessed SoVI to extreme precipitation under different recurrence intervals in Henan province based on three dimensions from EI, SI and RI, which concealed two laws of SoVI:

### 5.1 Spatial distribution law of SoVI to extreme precipitation in Henan province

The spatial distribution of SoVI in Henan province varied with the increase of recurrence intervals. Generally speaking, SoVI was low in the west and high in the north under recurrence intervals of 50a. High SoVI areas expanded to the middle and south as recurrence intervals increased.

The spatial distribution of SoVI in Henan province was dependent on EI, SI and RI in each area. High SoVI was mainly caused by high exposure, sensitivity and low resilience. For example, Anyang was the highest in SoVI due to its higher EI with day max precipitation ranking 4. In addition, the SI was moderate but RI was the lowest with average number of students in high school and highway passenger volume every ten thousands people far below the average. The three factors together made it most vulnerable to extreme precipitation. On the contrary, Low SoVI was determined by low EI, SI and high RI. For instance, Jiyuan, Luoyang, Sanmenxia and Jiaozuo were low in SoVI, which were attributed to the followings factors: First, EI in these areas were low, ranking the last four with day max precipitation far lower than the average 192.24mm/day. Second, SI were low in these areas ranking the last four. Among the indexes, population densities were lower than the average, with Jiyuan the lowest 0.9 person/km^2^. Proportion of old &young was around 32%, lower than the average 36.4%, the two of which mostly resulted to the low SI. Third, RI in these areas were all higher than the average, with Sanmenxia the highest. Among the indexes, number of hospital beds every ten thousands people, average disposable income of residents and highway passenger volume every ten thousands people were all much more than the average, which guaranteed the coping ability and recovery in emergency. In all, spatial distribution of SoVI was jointly determined by EI, SI and RI, through which countermeasures could be adopted to reduce SoVI. To begin with, prevention and mitigation measures should be taken in advance which included traditional land-altering structural approaches such as levees, flood walls, detention basins and green infra-structure, as well as nonstructural measures that removed people from risky areas like land use planning, elevating buildings [22]. Moreover, it was valid to eradicate illiteracy and poverty, increase awareness and construction of houses in safer or risk-free areas. Finally, it was fundamental to boost resilience by completing infrastructure such as educational, medical, transportation and communication facilities as well as disaster prevention and mitigation facilities, e.g. monitoring and alarming equipment, reservoir and drainage facilities.

### 5.2 Evolution law of SoVI to extreme precipitation in Henan province under different recurrence intervals

**Fig 8:**
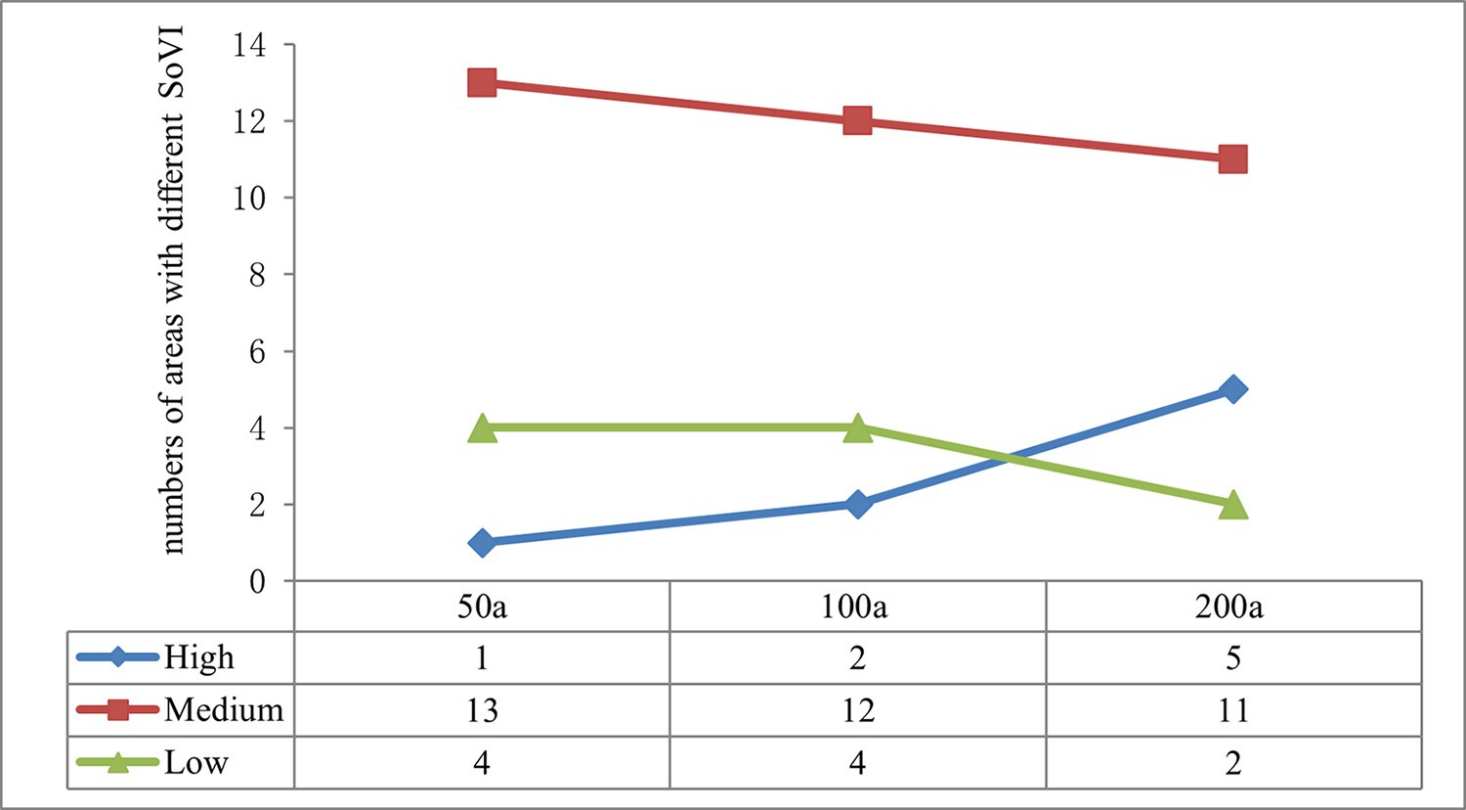
Evolution trend of SoVI to extreme precipitation in Henan province under three recurrence intervals. (Drawn by the authors themselves, the numbers of areas with different SoVI under three recurrence intervals are counted from Fig 7).

SoVI of each area and its spatial distributions both increased along with the recurrence intervals but with different growth rates, which gave rise to the evolution of SoVI under different recurrence intervals. As it is shown in Fig 8, When recurrence interval enlarged 2 times from 50a to100a, areas with high SoVI increased from 1 to 2, with the growth rate of 2. While high SoVI areas expanded from 2 to 5 at the rate of 2.5 after recurrence interval magnified 2 times from 100a to 200a, which was faster than the former. That is to say, the larger the recurrence interval is, the faster the SoVI increase. What underlies this law? On one hand, in geography, Henan is vast in area, stretching across the Yellow River, Huaihe River and Haihe River. The precipitation in each area varies greatly, which leads to the precipitation discrepancy under different recurrence intervals. On the other hand, in theory, SoVI is a function of EI, SI and RI, where EI is independent variable and grows with recurrence intervals, SI and RI are constants, SoVI is dependent variable, which will enhance as EI enlarges. While the growth rates of EI in each area under three recurrence intervals are distinct, the larger the recurrence interval is, the higher level the disaster is, which in turn leads to a higher exposure. As SI and RI in each area are stable during a short time period, so, the increase ratio of SoVI is mainly dependent on the growth rate of EI, which is positively related to recurrence intervals. The law also proved that social vulnerability is relative rather than absolute, it is a measurement of losses caused by a specific external shock such as extreme precipitation. Different types of disasters result in distinct losses. Despite the same type of disaster, losses vary if disasters occur at different times and with different magnitudes. That is to say, social vulnerabilities are diverse among different levels of disasters. Therefore, when making researches on the social vulnerabilities of disasters, we should consider not only social, economic, environmental and institutional factors, but also the specific type and levels of disasters, which are usually neglected by many scholars, who think that disasters in each area are identical, however, it is not the case. It is meaningless to make assessment on SoVI to marine disasters in inland areas or landslide &debris flows in plains as these calamities hardly occur in those areas. Similarly, it is unscientific to assess SoVI neglecting specific disaster magnitude or the exposure degree. Computing, comparing and analyzing SoVI under different extreme precipitation scenarios are just the very innovation of this study.

One of the findings in our study was that SI and RI hardly changed with recurrence intervals as the demographic characteristic, economy status and coping ability of the society remained constant during a time period, which resembled Eric Tate, et al [30] who made research on rainstorm exposure and social vulnerability in the United States, and finally concluded that the leading indicators and their rank order were robust to a change in the rainstorm disaster from the 100-year to the 500-year return period. It proves that the results in our present study are accurate and credible in some way. In another study on risk evaluation of natural disaster in Henan province [6], the results showed that Zhumadian, Shangqiu and Zhoukou were high in SoVI, Jiyuan and Sanmenxia were in low SoVI, which conforms with the social vulnerability distribution in our study.

The results in the study identifies where are socially vulnerable to extreme precipitation under different recurrence intervals, which are essential to tailor mitigation strategies. Nevertheless, several issues have been found in this study: First, the data in our study are mainly obtained from Statistic Yearbook, however, some of the data are not available, which makes difficulty in quantitative analysis. Second, the robustness of SoVI is contingent on the selection of variables, weighting schemes, size of the study area and aggregation methods. There is no consistent agreement on which indicators must be used to truly reflect the multidimensional and latent nature of vulnerability. The unevenness of the indicators will make a discrepancy on the results of social vulnerability, which also can be affected by weight assignment.

## 6. Conclusion

This paper took Henan province as an example and established indicators system of social vulnerability based on MOVE framework, Then, EI, SI, RI and SoVI were respectively calculated and mapped under three reoccurrence intervals. The main findings of the paper are as follows:

1. Social vulnerability was relative rather than absolute, which were diverse among different levels of disasters. We should consider not only social, economic, environmental and institutional factors, but also the specific type and levels of disasters.
2. SoVI in each area increased along with the recurrence intervals, but with different amplitude. The larger the recurrence interval was, the faster the SoVI increased.
3. SoVI was low in the west and high in the north. High SoVI areas expanded to the middle and south as recurrence intervals increased.

Although this study extends and enriches the research of social vulnerability both in research approach and indicator selecting, two aspects need to be explored in future researches: Firstly, in terms of methodology, multidimensional approaches, machine learning-based models, high-resolution satellite data, hydraulic model are suggested for in-depth rainstorm disasters analysis. Secondly, Loss caused by disasters is one of the important influencing factors of vulnerability. Therefore, personal casualties and economic losses of different disaster scenarios shall be incorporated into social vulnerability assessment in future research.

## Supporting information

S1 File Data. (ZIP)

## References

[1] Kolen B, Van Gelder PHAJM. Risk-Based Decision-Making for Evacuation in Case of Imminent Threat of Flooding. Water. 2018;10(10) 1429. 10.3390/w10101429

[2] Rana I.A., Routray J.K. Multidimensional Model for Vulnerability Assessment of Urban Flooding: An Empirical Study in Pakistan. Int J Disaster Risk Sci. 2018;9: 359–375. 10.1007/s13753-018-0179-4

[3] Rendani B.M, Agnes M, Nethengwe N. S. An assessment of flood vulnerability and adaptation: A case study of Hamutsha-Muungamunwe village, Makhado municipality. J DISAST RISK Stud. 2019,11(2):a692. 10.4102/jamba.v11i2.692 PMC6620490 31308887

[4] Cui KK, Liu DL. Li XH. Evaluation on social vulnerability to flood hazards in Henan section of Yellow River basin.B SOIL WATER CONSERV. 2021;41(5):304–310. 10.13961/j.cnki.stbctb.2021.05.039

[5] He SF L. Du LP, Gao XH. Assessment of social vulnerability to natural disasters on county scale in Henan province. R SOIL WATER CONSERV. 2015;22(6):293–297. 10.13869/j.cnki.rswc.2015.06.041

[6] Liu DL. Risk evaluation of flood disasters in Henan province based on GIS. B SOIL WATER CONSERV. 2014;34(3):126–129. 10.13961/j.cnki.stbctb.2014.03.024

[7] Zhang YL, You WJ. Social vulnerability to floods: a case study of Huaihe River Basin. Nat Hazards.2014; 71: 2113–2125. 10.1007/s11069-013-0996-0

[8] Nasiri H, Yusof M.J.M, Ali, T.A.M, et al. District flood vulnerability index: urban decision-making tool. Int. J. Environ. Sci. Technol. 2019;16:2249–2258. 10.1007/s13762-018-1797-5

[9] Birkmann J, Cardona O.D., Carreño M.L. et al. Framing vulnerability, risk and societal responses: the MOVE framework. Nat Hazards. 2013;67:193–211. 10.1007/s11069-013-0558-5

[10] Song J, Huang B, Li R Assessing local resilience to typhoon disasters: A case study in Nansha, Guangzhou. PLoS ONE. 2018;13(3): e0190701. doi: 10.1371/journal.pone.0190701 29522526 PMC5844519

[11] Eriksen C., Simon G.L., Roth F, et al. Rethinking the interplay between affluence and vulnerability to aid climate change adaptive capacity. Climatic Change. 2020;162:25–39. doi: 10.1007/s10584-020-02819-x 33184523 PMC7644517

[12] Hamidi A.R, Wang J, Guo S, et al. Flood vulnerability assessment using MOVE framework: a case study of the northern part of district Peshawar, Pakistan. Nat Hazards. 2020;101:385–408. 10.1007/s11069-020-03878-0

[13] Maiti S, Jha SK, Garai S, et al. An assessment of social vulnerability to climate change among the districts of Arunachal Pradesh, India. ECOL INDIC. 2017;77:105–113. 10.1016/j.ecolind.2017.02.006

[14] Zhang S, Zhang F, Wang C, Wang Z. Assessing the resilience of the belt and road countries and its spatial heterogeneity: A comprehensive approach. PLoS ONE. (2020;15(9): e0238475. doi: 10.1371/journal.pone.0238475 32877439 PMC7467326

[15] Belmonte C, Butrón A.M. Estimation of flood risk thresholds in Mediterranean areas using rainfall indicators: case study of Valencian Region (Spain). Nat Hazards. 2015;78: 1243–1266.

[16] Roncancioa D.J, Cutter S.L, Nardocci A.C. Social vulnerability in Colombia. Int. J. Disaster Risk Redu. 2020;50:101872. 10.1016/j.ijdrr.2020.101872

[17] Miller A.E, Steele N, Tobin B.W. Vulnerability and fragility risk indices for non-renewable resources. Environ Monit Assess. 2018;190(7): 373. 10.1007/s10661-018-6749-5 29860559

[18] Fuchs S, Keiler M, Glade T. Editorial to the special issue on resilience and vulnerability assessments in natural hazard and risk analysis. Nat. Hazards Earth Syst. Sci. 2017;17:1203–1206. 10.5194/nhess-17-1203-2017

[19] Jamshed A, Birkmann J, Feldmeyer D, Rana IA. A Conceptual Framework to Understand the Dynamics of Rural–Urban Linkages for Rural Flood Vulnerability. Sustainability.2020; 12(7):2894. 10.3390/su12072894

[20] Anderson C. C, Hagenlocher M, Renaud F. G. Comparing index-based vulnerability assessments in the Mississippi Delta: Implications of contrasting theories, indicators, and aggregation methodologies. Int. J. Disaster Risk Reduc. 2019;39:101128. 10.1016/j.ijdrr.2019.101128

[21] Debortoli N, Clark D.G., Ford J.D An integrative climate change vulnerability index for Arctic aviation and marine transportation. NAT COMMUN. 2019;10: 2596. doi: 10.1038/s41467-019-10347-1 31197167 PMC6565733

[22] Rathi SK, Chakraborty S, Mishra SK, Dutta A, Nanda L. A Heat Vulnerability Index: Spatial Patterns of Exposure, Sensitivity and Adaptive Capacity for Urbanites of Four Cities of India. INT J ENV RES PUB HE. 2022;19(1):283–299. 10.3390/ijerph19010283 PMC8750942 35010542

[23] Tate E, Rahman M.A, Emrich C. T, Sampson C. C. Flood exposure and social vulnerability in the United States. Nat Hazards. 2021;106: 435–457. 10.1007/s11069-020-04470-2

[24] Dintwa KF, Letamo G, Navaneetham K. Quantifying social vulnerability to natural hazards in Botswana: An application of cutter model. Int. J. Disaster Risk Reduc. 2019;37:101189. 10.1016/j.ijdrr.2019.101189

[25] Siagian TH, Purhadi P, Suhartono S, Ritonga H. Social vulnerability to natural hazards in Indonesia: driving factors and policy implications. Nat Hazards. 2014;70(2):1603–1617.

[26] Zhou Y, Li N, Wu W, Wu J. Assessment of provincial social vulnerability to natural disasters in China. Nat Hazards. 2014;71:2165–2186, 10.1007/s11069-013-1003-5

[27] Fekete A. Social vulnerability change assessment: monitoring longitudinal demographic indicators of disaster risk in Germany from 2005 to 2015. Nat Hazards. 2019;95(3):585–614.

[28] Godfrey A, Ciurean R.L, Westen C.J.van, Kingma N.C, Glade T. Assessing vulnerability of buildings to hydro-meteorological hazards using an expert based approach–An application in Nehoiu Valley, Romania. Int. J. Disaster Risk Reduc. 2015;13:229–241. 10.1016/j.ijdrr.2015.06.001

[29] Jamshed A, Rana I.A, Mirza U.M, Birkmann J. Assessing relationship between vulnerability and capacity: An empirical study on rural flooding in Pakistan. Int. J. Disaster Risk Reduc. 2019;36:101109–101120. 10.1016/j.ijdrr.2019.101109

[30] Tate E. Social vulnerability indices: a comparative assessment using uncertainty and sensitivity analysis. Nat Hazards. 2012;63: 325–347. 10.1007/s11069-012-0152-2

